# Hearing and early life adversity: effects of developmental stress on sensory processing

**DOI:** 10.1038/s41386-025-02203-2

**Published:** 2025-08-21

**Authors:** Merri J. Rosen, Julia J. Huyck

**Affiliations:** 1 Hearing Research Group, Department of Biomedical Sciences, Northeast Ohio Medical University, Rootstown, OH, USA.; 2 University Hospitals - NEOMED Hearing Research Center, Northeast Ohio Medical University, Rootstown, OH, USA.; 3 Brain Health Research Institute, Kent State University, Kent, OH, USA.; 4 Speech Pathology and Audiology Program, Kent State University, Kent, OH, USA.

## Abstract

In children, early hearing loss can cause prolonged difficulty with auditory perception and language processing. Yet children with hearing loss are at greater risk of long-term language, cognitive, and socioemotional deficits when raised with environmental challenges that are stressful, such as low socio-economic status. The neural circuits underlying language and auditory processing are shaped by auditory experience over the course of development, allowing listeners to make sense of environmental sounds including speech. Evidence is accumulating from work in animal models that these sensory circuits are also affected by adverse stressful experiences early in life. Recent experiments indicate that stress can exacerbate sensory deficits caused by developmental hearing loss. These effects are driven by shifts in mechanisms underlying developmental plasticity, as well as by consequences of altered activity of the hypothalamic-adrenal-pituitary (HPA) stress axis. Viewed through an interdisciplinary lens, the research reviewed here suggests that some of the challenges experienced by children with hearing loss may be intensified by early life adversity and ameliorated by interventions that target both sensory deprivation and stress.

## INTRODUCTION

In both humans and animals, it is well established that experiences during early life alter the central nervous system (CNS). In animal models of early hearing loss, perceptual problems are correlated with compromised sound encoding within the central auditory system [1–5]. Early hearing loss (HL) in children, even when transiently induced by chronic ear infections (i.e., otitis media), can induce long-lasting problems in auditory perception and language processing [6–10]. When children with even intermittent HL are raised with additional challenges such as low socioeconomic status (SES), they are at greater risk for long-term language deficits and socioemotional issues [11–14]. This higher risk could stem from any or all of three key dimensions of early life stress (ELS): material deprivation (i.e, lack of meeting basic physical or social needs), threat (i.e., presence of experiences that threaten physical or psychological integrity), or unpredictability [15, 16]. This raises the possibility that early-life stress may contribute to sensory deficits.

In this review, we draw connections across fields that suggest an interaction between detrimental early life experiences and hearing loss in humans, with mechanisms elucidated by studies in animals. We first review the literature on stress, adverse childhood experiences, and hearing loss in children. We then review neural mechanisms of stress and examine the evidence that ELS affects sensory systems in addition to its well-known detriments to emotion and cognition. We explore the neural mechanisms by which ELS may affect sensory regions, and evidence suggesting interactions between early stress and early hearing loss. Finally, we suggest approaches for prevention and intervention to mitigate poor outcomes in deaf and hard-of-hearing (DHH) children.

## RATIONALE FOR EXAMINING EFFECTS OF EARLY-LIFE EXPERIENCE ON SENSORY REGIONS

Early development provides an opportunity for our nervous systems to adapt to the world around us, shaped by the milieu of sensory stimuli which comprise the environment we will need to interpret and navigate throughout life. As sensory systems develop, they require input to help establish and refine processing circuitry, which is enabled by mechanisms of heightened plasticity in the CNS. Yet this enhanced plasticity is a double-edged sword, where the ability to be optimized by experience means that sensory deprivation or maladaptive experiences can cause enduring deficiencies in sensory processing skills. This is well-studied in terms of sensory perception and extends across sensory modalities. For example, both the perception and cortical encoding of temporally-varying sounds such as speech mature gradually to reach adult performance levels, providing an extended postnatal window of susceptibility to altered auditory experience [17–19]. During postnatal critical or sensitive periods of heightened development within a larger window of gradual maturation, atypical sensory experience such as developmental hearing loss can lead to persistent auditory perceptual deficits, accompanied by changes in auditory cortex (an effect originally demonstrated by visual deficits arising from altered visual cortical development) [2, 20–25]. In humans, auditory critical periods are often viewed through the lens of language acquisition, with congenital hearing loss having increasingly severe and longer-lasting effects on language ability as it remains untreated for a longer period of time [26]. Data from humans indicate that sensory deprivation such as hearing loss is associated with impaired socioemotional functioning. For example, DHH children who have difficulty understanding speech experience poorer communication, more attentional problems, and increased anxiety and depression [27].

Like sensory development, emotional regulation and cognition are also shaped by early experiences, as demonstrated by correlational studies in humans and supported by early-life manipulations in animals. In particular, adverse early life experiences are well known to increase the risk of later anxiety and depressive disorders, as well as affecting attention, learning, memory, arousal, and emotional self-regulation [28–35]. This is driven by changes in brain regions responsible for emotion and higher-level cognitive functions, including the amygdala, hippocampus, and prefrontal cortex. Threat, material and social deprivation, and unpredictability are three dimensions of childhood stress that may lead to different behavioral and biological outcomes. For example, though anxiety and depression have been associated with all three dimensions of early life adversity [36–40], threat might be more strongly associated with anxiety [41], problem behaviors [42], and emotional reactivity [43]. Further, threat has been shown to alter patterns of latent trait cortisol while deprivation does not [44]. Similarly, deprivation may have greater health and economic consequences than other dimensions of developmental stress [36] and may be more strongly predictive of cognition and academic success [42, 43]. The cortical differences associated with ELS seem to be particularly sensitive to mitigating factors related to the amount of material and social deprivation such as family income and high-quality caregiving experiences [45, 46]. Finally, unpredictability may mediate some of the behavioral sequelae of threat and material and social deprivation (e.g., ref. [44]), and may be associated with distinct changes in functional connectivity [38].

A commonality between ELS-related detrimental changes to emotional and cognitive regions and sensory deprivation-related detrimental changes to sensory systems is atypical experience during critical or sensitive periods of development. Indeed, a broad meta-analysis of childhood adversity indicates a sensitive period framework, where the developmental timing of adversity best accounts for influences of early adverse experiences [47, 48]. Despite this common thread, investigations of developmental brain plasticity have typically been siloed: effects of sensory deprivation or enrichment are examined in primary sensory regions such as auditory or visual cortex, while effects of stress are examined in emotional and cognitive regions such as amygdala or prefrontal cortex. Yet developmental windows of heightened critical period plasticity across brain regions are enabled by a common suite of molecular elements [49]. While these critical period elements and their role in plasticity were originally elucidated in visual cortical regions, they are also at play within brain regions involved in emotional regulation and cognition that are known to be susceptible to early-life stress. Thus ELS-induced deficits in these behavioral functions are accompanied by shifts in critical period elements within the amygdala, hippocampus, and prefrontal cortex [50–53]. Yet the effects of ELS on sensory regions have only recently received attention. Furthermore, ELS consequences are commonly mediated by stress hormones and downstream effects involving stress receptors. The presence of such receptors in sensory regions is a potential indicator of direct effects of ELS on sensory processing.

Decades of research on ELS in both humans and animals have focused on emotion and higher-order cognitive functioning [35, 50, 54–57]. More recently, animal studies are increasingly providing evidence that ELS negatively affects sensory regions and sensory perception. Deficits in sensory encoding may worsen attentional, cognitive, and emotional problems induced by ELS. Sensory systems are linked through both direct and indirect pathways with brain structures involved in cognitive and emotional functions [58–62]. Thus if sensory representations are degraded, the fidelity of sensory information available to higher neural regions known to be altered by ELS will be compromised, contributing to behavioral dysregulation. For example, paying attention to speech in a classroom would be particularly difficult and frustrating if auditory signals are poorly encoded even before reaching higher regions.

Given that both sensory deprivation and early life adversity may affect sensory encoding, cognitive functioning, and emotional regulation, it stands to reason that the combination of these two factors may have especially detrimental effects. Indeed, among DHH children, cognitive, linguistic, and socioemotional outcomes are poorer among individuals who have lower socioeconomic status [11, 63–73], which is sometimes used as a proxy for early life adversity, as discussed below.

## CONCEPTUAL FRAMEWORK

**Fig. 1:**
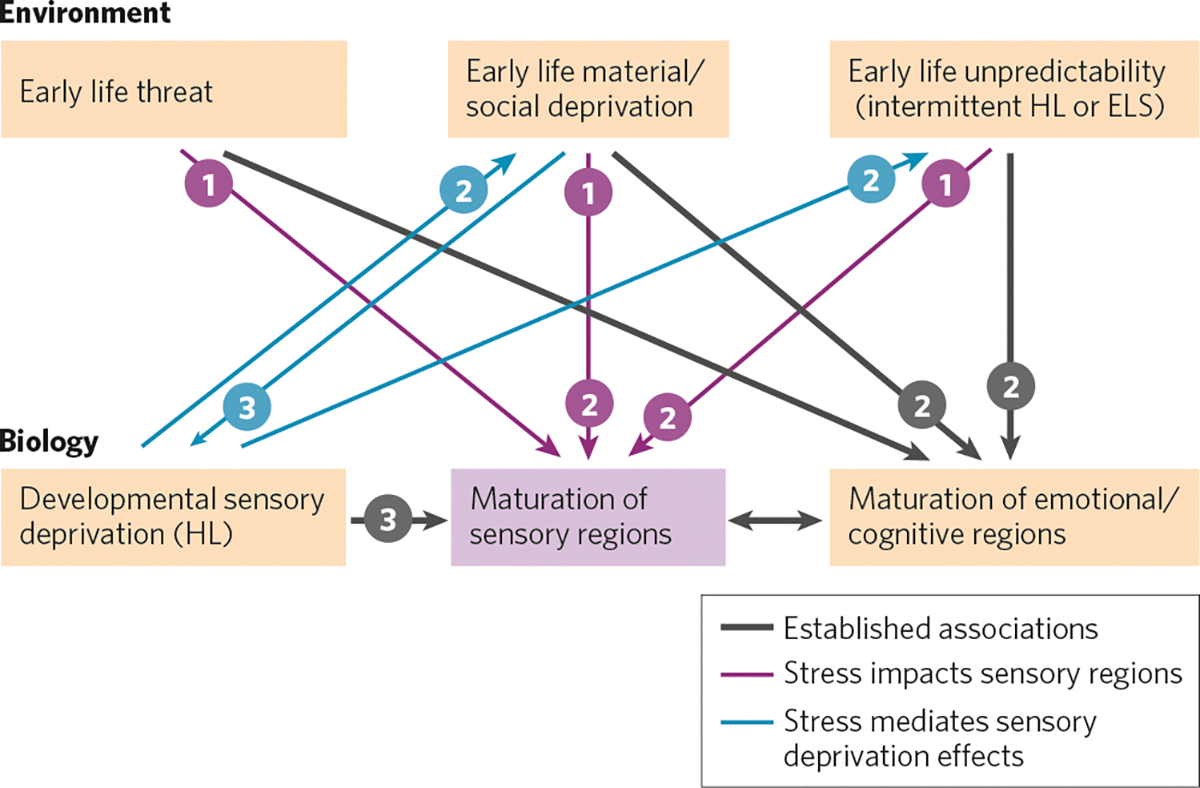
A conceptual framework for understanding the effects of early life stress and developmental sensory deprivation on maturation of brain regions responsible for sensory function. *Numbers* indicate interactions involved in each of three elements of the framework (see text). Well-established associations are in *thick black lines*. *Magenta lines* indicate a proposed direct impact of three dimensions of stress on maturation of sensory areas. *Blue lines* indicate interrelationships that allow for dimensions of stress to mediate the effects of developmental sensory deprivation on the maturation of sensory areas of the brain.

It is well-established that sensory deprivation disrupts maturation of sensory regions of the brain and that dimensions of early life stress (i.e., threat, material/social deprivation, and unpredictability) affect maturation in areas of the brain that underlie emotions and cognition (Fig. 1, *thick black lines*). Here we propose a framework with three additional Elements. Element 1) Early life stress via all three dimensions may directly influence maturation of sensory regions through some of the same mechanisms by which stress affects emotional and cognitive regions of the brain (Fig. 1, *magenta lines*). Element 2) Because sensory deprivation can lead to social and linguistic deprivation and may result in unpredictable interactions with the environment, these types of ELS may also mediate the effects of sensory deprivation on both sensory development and emotional/cognitive development (Fig. 1, *lines labeled* ‘*2*’). Element 3) Poor communication in deprived households could reduce sensory input, thus contributing to sensory deprivation. In addition, material deprivation during childhood may lead to prolonged periods of untreated sensory deprivation (e.g., hearing loss), which in turn will affect maturation of sensory regions (Fig. 1, *lines labeled* ‘*3*’). Throughout this manuscript, we draw attention to studies that provide support for the three Elements depicted here.

## HEARING LOSS AND STRESS IN CHILDREN

While there have been few studies directly addressing the relationship between early life stress and hearing loss in human populations, there is considerable evidence that hearing loss itself might be a source of stress in some children (Fig. 1, blue lines, *Element 2*). Most of these studies have focused on long-term hearing loss rather than temporary or intermittent losses. DHH children with mild to moderate hearing loss exhibit higher cortisol levels that may be attributable to increased listening effort [74]. Levels of listening-related fatigue can be high in DHH children with moderate to profound hearing loss [75, 76] and may be a greater issue than their parents or caregivers realize [75]. DHH children with a variety of losses have also been shown to have higher levels of psychiatric and socioemotional problems than their normal-hearing peers [72, 73, 77–81]. Suggesting that social and linguistic deprivation may play a critical, mediating, role in these outcomes (Fig. 1, *Element 2*), DHH children are less engaged with their peers and school communities [82] and DHH children who have difficulty communicating are especially prone to mental and behavioral health issues [27, 71].

Families of DHH children may experience higher levels of caregiver stress (i.e., stress experienced by parents or other caregivers) due to a combination of hearing loss specific variables (e.g., age of identification and amplification) and child-related characteristics (e.g., additional disabilities, child’s socioemotional health), as well as factors related to perceived competence and social support (e.g., general social support, hearing loss caregiver self-efficacy, hearing loss-related support) [83–87]. Several studies have demonstrated a correlation between caregiver stress and cognitive, language, and behavioral outcomes in DHH children; however, the causal relationship among these factors has not been established. Among DHH children with mild to profound losses, increased levels of caregiver stress or more negative parental sensitivity (a measure of a parent’s views towards their child) are associated with poorer language ability [85–88] and social communication [85, 86, 89]. Some studies propose models in which issues with communication directly or indirectly increase caregiver stress [83, 89], which could then affect sensory and emotional/cognitive maturation (Fig. 1, *Element 2)*. Other models suggest that higher caregiver or family stress directly or indirectly leads to poorer communication [87, 88], which could contribute to social and sensory deprivation and affect sensory, emotional, and cognitive development (Fig. 1, *Elements 2 and 3*). Even among normal-hearing (NH) children, higher levels of caregiver stress are associated with fewer child vocalizations and conversational turns at age 6–12 months [90], differences in baseline brain activity as measured with electroencephalography [90], and altered neural responses during auditory statistical learning at age 26 months [91]. Among DHH children, higher caregiver stress is associated with poorer inhibitory and attentional control [83, 88, 89] and greater socioemotional and behavioral health issues [83, 84, 89]. Social deprivation may not be the only factor contributing to an interrelationship between caregiver stress and emotional/cognitive outcomes because these associations persist even when DHH and normal-hearing children have similar language abilities [92]. Suggesting that the combination of hearing loss and high caregiver stress might be more detrimental than stress alone (Fig. 1, *Elements 2 and 3*), the association between caregiver stress and these cognitive, language, and behavioral measures persists in DHH children but not NH children when the two groups report similar levels of caregiver stress [88].

While human studies are often correlational, animal models have demonstrated causal relationships between early sensory experience and cognitive impairments. Animals raised in enclosures with constant audiovisual stimulation designed to mimic television exposure but not induce stress displayed later alterations in cognition, anxiety, and risk-taking behavior [93, 94]. On the flip side of sensory stimulation, transient HL during development induces deficits in perceptual learning, a cognitive process involving the orbitofrontal cortex [95–97]. As these types of experiments do not manipulate stress, future work extending these approaches is needed to determine the role that ELS and HL interactions have on higher-order function.

## SOCIOECONOMIC STATUS AS A PROXY FOR STRESS IN HUMANS

Due to the relative scarcity of studies directly examining the combined effects of hearing loss and stress in humans, we will also consider the interaction of hearing loss and socioeconomic status (SES) during development. Low SES is an imperfect but reasonable proxy for early life stress or childhood adversity because low-income and racially-minoritized families typically experience higher levels of self-reported stress [98] and a greater number and higher intensity of stressful life events such as marital distress, parental incarceration, exposure to violence, involvement in the foster care system, food insecurity, and housing instability [99–102]. Hair cortisol concentration is a commonly used measure of chronic stress that assesses the HPA axis [103] and has been shown to be elevated in children from families with low parental education, but not necessarily low familial income [104, 105]. However, the relationship between hair cortisol and chronic stressors may be complex: In a longitudinal study across childhood and adolescence, both lower and higher levels of cumulative lifetime adversity (a composite measure including low SES, maternal alcohol use hostile-reactive parenting, maternal depression, peer victimization, and neighborhood dangerousness) were associated with higher hair cortisol [106]. One factor contributing to this non-linear effect may be that certain stressors, like child maltreatment, lead to reduced rather than elevated hair cortisol [107]. In addition, cortisol responses to stress may also vary with race. One study found that perceptions of unsafety were linked with higher hair cortisol concentrations, but only among Black and not white adolescents [108]. Another study reported that high neighborhood stressor exposure was associated with increased serum cortisol, a measure of both acute and chronic stress, in children of European descent but with decreased serum cortisol in children of African descent [109]. Though these results are somewhat mixed (for a review see [110]), together they build the case that measures of socioeconomic status may relate to HPA axis activation and stress in children and adolescents.

Given the interconnection between low SES and high stress, here we discuss the relationship between SES (typically familial income and/or parental education) and hearing outcomes. While this relationship, like the one between SES and stress, is likely multifactorial, there are three main ways that SES and hearing have been shown to interact: (1) children with lower SES are more likely to have untreated hearing loss and therefore experience more sensory deprivation (Fig. 1, *Element 3*), (2) lower SES is associated with altered auditory processing (Fig. 1, *Element 1*), and (3) SES is predictive of language, literacy, and behavioral health outcomes in children both with and without hearing loss (Fig. 1, *Elements 1 and 2*).

## SOCIOECONOMIC STATUS AND UNTREATED HEARING LOSS

One way that socioeconomic status and hearing loss are related is that individuals from lower SES backgrounds are more likely to have untreated hearing loss (Fig. 1, *Element 3*). Hearing loss (HL) affects ~1 to 2 out of every 1000 (0.1–0.2%) newborns [111, 112] and impacts increasing numbers of children throughout childhood, reaching nearly 20% by adolescence [113]. Increased prevalence rates of permanent pediatric sensorineural hearing loss (SNHL) have been associated with lower familial income [113, 114], residence in urban low-income neighborhoods [115], and lower maternal education [116]. Other socioeconomic factors contributing higher prevalence of permanent SNHL include minority race/maternal country of origin [115–117], male gender [116, 117] and exposure to higher levels of environmental and community noise. One common cause of intermittent conductive hearing loss (CHL) in young children is recurrent otitis media with effusion (i.e., ear infections with fluid in the middle ear). These ear infections are more common in lower SES children [14, 118–120]. The higher prevalence of ear infections in lower SES children has been associated with stress, overcrowding in the home, contact with large numbers of other children, exposure to cigarette smoke, and reduced access to specialty health care [14, 118, 120–123]. The intermittent CHL that can result from chronic ear infections is associated with a higher risk of permanent SNHL loss later in life [124] as well as long-lasting problems in auditory perception and language processing in some children without permanent HL [9, 12], perhaps due to the inherent unpredictability of intermittent HL (Fig. 1, *Element 2*, specifically the arrows from early life unpredictability to sensory and cognitive/emotional development).

Not only is the prevalence of pediatric hearing loss greater in populations with lower SES, but individuals from low SES communities also may take longer to be treated for chronic otitis media with effusion [118, 119, 125] and have delayed access to hearing health care [119, 126]. Even once hearing loss has been identified, families with lower SES are less likely to follow up with appointments after their child failed to pass their newborn hearing screening [127–129]. Children with lower SES also may receive amplification (i.e., hearing aids or cochlear implants) at a later age [130] and experience more post-operative complications following cochlear implantation. Following amplification, they may be less likely to attend follow up appointments [118, 127, 131–133] or to receive regular rehabilitative therapy such as speech therapy [129, 130, 134].

## SOCIOECONOMIC STATUS AND AUDITORY PROCESSING

The detrimental effects of lower SES on cognition, language, and academic performance are well documented and beyond the scope of this paper, with higher stress and fewer, less complex, language interactions frequently considered as contributing factors (for reviews see [135, 136]). In addition to these considerations, SES- or stress-related differences in auditory processing may contribute to cognitive, linguistic, and academic outcomes as well.

Children who come from lower SES backgrounds demonstrate impaired sound discrimination and listening skills on speech and nonspeech tasks compared to higher SES peers (Fig. 1, *Element 1*). Several imaging studies have shown a relationship between SES and auditory speech and nonspeech processing as measured using magnetoencephalography (MEG), electroencephalography (EEG), or functional magnetic resonance imaging (fMRI). Findings include SES-related deficits in auditory selective attention among 3- to 8-year-olds, as indicated by similar EEG refractory periods for attended and unattended stimuli [137], and slower processing speed or poorer detection of stimulus changes among 6- to 12-year-olds whose parents reported lower SES, as shown by later MEG P300 responses to auditory oddball words and nonwords [138]. High schoolers with lower maternal SES also demonstrate noisier neural activity both in the absence of auditory stimulation and in response to speech and weaker EEG responses to speech stimuli than peers matched for age, ethnicity, and school [139]. The effects of SES can also interact with task performance, such that differences in SES are only apparent for children who are having more difficulty with a listening task: During a phoneme categorization task, maternal education predicted activation in the left prefrontal cortex, but only for children who were less proficient at the task [140]. This result is consistent with behavioral data showing impaired speech perception and phonemic awareness in children from lower SES families, with children of lower SES demonstrating similar performance to those with histories of chronic otitis media [11, 64]. Because lower SES is sometimes a predictor of poorer auditory processing in the absence of hearing loss, it is possible that hearing loss and low SES both have direct effects on auditory processing but that these effects do not interact.

## SOCIOECONOMIC STATUS AND OUTCOMES OF HEARING LOSS

Language, literacy, and behavioral outcomes for DHH children correlate not only with caregiver stress, as reviewed above, but also with SES (Fig. 1, *Elements 2 and 3*). Behavioral health problems including attention deficit hyperactivity disorder, anxiety, and conduct disorder are correlated with socioeconomic status among DHH children with long-term losses [71–73]. Poorer speech perception, receptive language, expressive language, and literacy have also been associated with lower SES, caregiver education, and access to family resources for DHH children with both intermittent and long-term losses [11, 63–70]. These effects seem to be mitigated, at least somewhat, by the provision of appropriate speech and language therapy and high-quality caregiver-child communicative interactions [134, 141–143], suggesting a mediating role of social environment (Fig. 1, *Element 2*).

Among DHH children, one of the main predictors of speech and language is early detection and intervention [144–147]. Given that lower SES children are more likely to have delayed treatment for hearing loss, including speech therapy, the poorer language and reading scores of low-SES children with hearing loss could be due, in part, to later identification and intervention. Another possible contributor is that the quality of the language environment and caregiver interactions may be poorer, on average, in lower SES homes [148–153] (Fig. 1, *Element 3*).

Although SES is used as a proxy for stress, studies in children have not evaluated auditory processing deficits that are unambiguously tied to early-life stress. As detailed below, animal studies have elucidated mechanisms by which early adverse experiences such as stress and hearing loss may cause neural changes and behavioral challenges. To lay the groundwork for this discussion, we briefly review the mechanisms by which stress affects neural elements and outcomes.

## NEURAL MECHANISMS OF EARLY-LIFE STRESS

Adverse early life experiences have long lasting effects via chronic dysregulation of the HPA axis, a major neuroendocrine system that adapts organisms to environmental change and regulates processes throughout the brain and body. The HPA axis involves feedforward signaling via release of corticotropin releasing factor (CRF) and arginine vasopressin (AVP) from the hypothalamus, which activates the pituitary to signal release of corticosteroids (CORT) from the adrenal glands (primarily cortisol in humans and corticosterone in rodents). Following acute stressors, negative feedback from elevated CORT levels regulates hypothalamic activity to normalize the stress response via activation of glucocorticoid (GR) and mineralocorticoid receptors (MR) [154]. While this functions well to adapt organisms to acute stressors, it is maladaptive when chronically activated. Chronic activation can subsequently induce a wide range of effects on genes related to development via epigenetic modification [155–160].

Chronic activation of the HPA axis induces dysregulation indicated by altered circulating levels of CORT. Early-life stress, which often involves altered caregiving by the mother, causes dysregulation of the HPA axis, resulting in either increased or decreased circulating levels of CORT and enhanced or blunted stress responses [161–165]. GR and MR expression in neural regions is directly influenced by CORT, such that cells down- or upregulate expression levels in the presence of high or low levels of CORT, respectively [166–168]. Importantly, this has functional effects on neural processing, as MR or GR activation alter neuron excitability [169–175]. Furthermore, CRF functions to modulate synaptic transmission and plasticity, in addition to its role in altering levels of circulating CORT [176–179]. Thus HPA axis dysregulation can influence levels of glucocorticoid hormones and receptors to alter neural circuitry and behavior.

Effects of ELS vary depending on neural maturation. Based on synaptogenesis and subsequent synapse elimination indicating heightened plasticity, developmental time windows of maturation occur earlier in sensory regions (e.g., auditory and visual cortices) than in higher-order regions involving emotional regulation and cognition (e.g., prefrontal cortex) [180–182]. Sensory and prefrontal regions are interconnected [58–62], and this maturational sequence suggests that adverse sensory experience can impact higher-order functions [97, 183–185]. This timeline would allow altered auditory processing to induce social and linguistic deprivation and unpredictable interactions with the environment, contributing to ELS-induced emotional and cognitive changes (Fig. 1, *Element 2*). The idea is supported by a recent analyis of children with low SES in the Adolescent Brain Cognitive Development (ABCD) study, where reduced thickness in prefrontal cortex was mediated by structural differences in sensory regions [186]. However, synaptic elimination and plasticity extend well into adolescence in both auditory and emotional regions (e.g., refs. [19, 182, 187]), providing an extended time period where the number and strength of synapses are malleable by adaptive or adverse experiences, and where sensory and emotional experiences could have broad impacts.

Within this window of gradual maturation, regions within the developing brain have unique critical or sensitive periods where they are most vulnerable to disruption by sensory experiences [28, 188–191]. Animal studies manipulating visual or auditory input have elucidated the mechanisms underlying these critical periods of development, which have been studied primarily in visual and auditory cortices. Yet the mechanisms underlying sensory critical periods also shape maturation in non-sensory brain regions, and there is evidence for critical periods in regions susceptible to early-life stress. For example, fear extinction learning emerges between P17 and P24 in rats, a window that is correlated with critical period closure in the amygdala indicated by shifted inhibitory levels and increased synaptic stability [192, 193]. Multiple distinct critical periods throughout development exist, both for different aspects of auditory processing such as frequency tuning, temporal modulation sensitivity, or frequency modulation (FM) encoding [17, 194–197], as well as for different aspects of emotion such as anxiety, depression, and aggression [198–200]. This highlights the complexity of possible interactions of auditory and emotional processing based on the developmental timing of ELS.

While a full description is beyond the scope of this review, there are several critical period elements commonly involved with both ELS and sensory deprivation (such as that accompanying hearing loss). The opening, maintenance, and closure of sensory critical periods are characterized by shifts in multiple molecular elements including excitatory-inhibitory balance, inhibitory parvalbumin (PV) neurons, perineuronal nets (PNNs), and brain derived neurotrophic factor (BDNF) (reviewed in ref. [25]). Upregulation of PV neurons is triggered by BDNF and is necessary for critical period opening, while PNNs are extracellular matrix molecules that form primarily around inhibitory neurons and close critical periods by limiting synapse formation. These elements are sensitive to early experience. Shifts in these critical period elements are induced by sensory deprivation in auditory, visual, and somatosensory cortices, and coincide with perceptual deficits [190]. Similarly, these critical period elements are altered by ELS in prefrontal cortex, amygdala, and hippocampus in conjunction with emotional and cognitive deficits [50, 51, 201–204]. Because critical period elements affected by stress in higher regions are common to sensory regions, this suggests that sensory regions and perception should be altered not only by modality-specific perturbations such as hearing loss, but also by adverse experiences such as stress.

Furthermore, interactions between the timing and nature of stress induction are important. Early sensory experiences are particularly influential when they involve elements of stress including threat (e.g., due to fragmented maternal interactions, violence, or abuse) or material or social deprivation (e.g., the loss of expected sensory stimulation due to neglect or institutional rearing). Early threat has its greatest effects on emotional brain networks involving the amygdala, hippocampus, and prefrontal cortex [205–208]. In contrast, early life deprivation is generally associated with reduced cortical volume and synaptic complexity, particularly in sensory regions [208–212]. Thus, ELS effects on plasticity and the HPA axis vary based on brain regions, as well as on elements including behavior examined, details of stress induction (e.g., type of stressor, animal age at induction, duration and unpredictability of stress), and individual genetic susceptibility [213, 214]. Depending on the type of stress, ELS can shift the timing of critical periods, thus changing susceptibility to subsequent experience [202, 215–218]. ELS associated with threat *accelerates* maturation in frontal cortex, amygdala, and hippocampus based on the developmental timing of specific behavioral responses and levels of PV neurons and PNNs [202, 208, 219–221]. In contrast, sensory deprivation often *delays* critical periods, extending the time window in which sensory experience induces plasticity [222–226]. Childhood institutionalization, which involves profound deprivation, is associated with language delays and sensory processing challenges [227–229], supporting the idea that material or social deprivation also induces critical period delays [15]. As a less extreme example, the Child Development Project found an association between early deprivation and lower verbal abilities in adolescence [230, 231].

Because brain regions mature at various postnatal time points, stress and other sensory perturbations will be particularly disruptive to specific brain regions and behaviors when induced during critical or sensitive periods for those regions. Mechanistically, this arises due to the increased plasticity during these periods, and the molecular brakes following these periods that preserve resulting changes. Thus the effects of ELS on sensory systems may be most dramatic when induced during critical periods for those sensory systems [232]. As an example, depending on its time window of induction, ELS has opposite effects on synaptic distribution in cingulate vs somatosensory cortex [233]. This may be due to differential maturational stages of these regions relative to their critical periods, as well as to effects driven by altered levels of neuroendocrine hormones and their receptors.

## DEFICITS IN SENSORY PERCEPTION AND SENSORY REGIONS ARISING FROM ELS

Most studies of ELS in humans focus on the effects that induce noticeable behavioral challenges following early adversity: anxiety, depression, emotional dysregulation, and cognitive dysfunction [35, 54, 55]. These studies generally take neuropsychological approaches rather than psychophysical approaches, resulting in a dearth of data on purely sensory behavioral deficits. Yet while studies evaluating how ELS affects sensory function (Fig. 1, *Element 1*) have been sparse, they are suggestive of sensory deficits. Childhood maltreatment changed gray matter volume in auditory, visual, and somatosensory cortices [234]. Children who experienced maltreatment had decreased volume of the left visual cortex [235] along with a reduction in the white matter tract connecting visual cortex with the amygdala [236]. Neglect also reduced visual attention and visual memory in children [237]. In infants who experienced recurrent crying spells, the maturation of visual evoked potentials was delayed, suggesting that early stress can hinder the development of visual perception [238]. Whether sensory dysfunction can be induced by ELS is being directly tested in several animal models.

Animal models of ELS allow control over the timing, type, and intensity of stress induction, enabling a better understanding of its behavioral outcomes and neural underpinnings. Like studies in humans, most animal studies of ELS focus on emotion and cognition. Yet animal studies are increasingly providing evidence that ELS affects sensory regions and sensory perception (Fig. 1, *Element 1*). A common method of early-life stress induction is maternal separation (MS) and isolation prior to weaning. This powerful stressor which deprives pups of sensory stimulation from maternal and sibling interactions falls under the categories of both deprivation and threat [15]. MS studies examining either visual or somatosensory function in animals found behavioral deficits in both sensory modalities that varied across studies. Various maternal separation protocols in rodents either caused enhanced sensitivity to limb stimulation or painful stimuli, or instead reduced responses in somatosensory cortex and impaired performance on whisker sensitivity tasks [239–241]. These behavioral deficits were rescued by blocking the activity of glucocorticoid receptors during stress induction [241]. In rodent somatosensory cortex, MS either reduced or increased dendritic length and spine density depending on animal age and duration of MS induction, and shifted excitatory and inhibitory function [233, 239, 241–243]. These varied effects may be due to differences in the timing of stress induction and species examined (rats vs mice) across these studies.

Kittens are the classic model for critical period ocular dominance plasticity in visual cortex. An early study demonstrated that cortisol injections during this critical period reduced plasticity in a dose-dependent manner, suggesting a potential role for ELS effects in visual cortex [244]. Studies of ELS on visual function in rodents did not use MS, but showed that dysfunction varied with the developmental time point at which stress was induced. Stress induced by shipping mice when they were postnatal day (P) 12 (a manipulation involving threat and unpredictability) caused *accelerated* visual development. This was indicated by early eye opening, premature visual acuity development, and an early closure of the critical periods based on early formation of PNNs [215]. In contrast, chronic mild unpredictable stress in the home cage (e.g., cage tilting, damp bedding) administered from P2–8 for 1 h daily in mice, did not alter visual acuity. Instead, it *delayed* the developmental critical period of plastic responses to monocular deprivation, preventing early plastic changes and extending the time window of plasticity. These effects were correlated with reduced inhibition, and were rescued by experimentally increasing inhibitory function during stress induction [218]. Furthermore in female mice, ELS extended this plasticity into adulthood, well past the normal closure of the critical period. This extended plasticity was driven by shifts in both CRF within visual cortex and CRF1 receptors on subsets of visual cortical inhibitory neurons [245]. This suggests that HPA axis dysregulation affects visual cortical function.

## EARLY-LIFE STRESS AND AUDITORY PROCESSING

In children, there is clear evidence for negative effects on auditory perceptual abilities by early life adversity (Fig. 1, *Element 1*). Early neglect reduced auditory attention, language, verbal memory, and verbal learning skills in children [237, 246], and prenatal maternal stress altered the development of speech perception in infants [247]. These behavioral changes are reflected in auditory cortical and even subcortical effects. Early adversity is associated with decreased cortical thickness in the superior temporal sulcus, and exposure to parental verbal abuse is associated with increased gray matter volume in the superior temporal gyrus [40, 248]. A longitudinal study that examined brain regions in adolescents before and after the Covid-19 pandemic noted accelerated brain maturation based on cortical thickness in the left superior temporal gyrus [249].

Animal studies are corroborating these results and providing both correlational and causal evidence for mechanistic changes in auditory brain regions underlying impaired auditory perception. In macaques, maternal deprivation caused precocious myelination in the left superior temporal sulcus [250], similar to the anatomical changes found in humans. We recently tested the effects of ELS on Mongolian gerbils, a well-established model for auditory processing in which a developmental critical period for the auditory cortex (ACx) has been established [18]. This critical period begins immediately after ear opening (~P10) and is a window of susceptibility, where hearing loss alters intrinsic and synaptic properties of ACx neurons, impairs auditory perception, and degrades ACx encoding of time-varying sounds [2–5, 251–253]. Intermittent unpredictable maternal separation during this ACx critical period in gerbils (a manipulation involving threat, deprivation, and unpredictability) impaired both behavioral detection and ACx responses to rapid sounds (short gaps in background noise), and diminished ACx responses to simple sounds [254, 255]. This is particularly relevant because the ability to detect rapid changes in sound (temporal processing) is intrinsic to deciphering our soundscape, including analyzing auditory scenes and understanding speech [256]. In a more naturalistic test, this ELS induction affected gerbil preference for contact calls and alarm calls [257]. Consistent with the idea that ELS induces its effect at least in part through altered critical period mechanisms, ACx in these animals had fewer inhibitory parvalbumin (PV) neurons and fewer PNNs [258]. ELS may also act directly within ACx following HPA axis dysregulation, as glucocorticoid receptors are known to be present in ACx [259, 260].

Others have confirmed and extended these results in rats, using an MS that begins well before ear opening (P2) but extends through the ACx critical period. Following MS, adult rats had impaired performance on behavioral tests of FM (frequency modulation) discrimination and sound azimuth detection, along with reduced selectivity and sensitivity to those sounds in ACx [261, 262]. These deficits were accompanied by changes in elements involved in critical period plasticity: fewer inhibitory PV neurons, fewer PNNs, and reduced brain derived neurotrophic factor (BDNF). An et al. also provided evidence of epigenetic modification driving the stress effects. When rat pups were exposed to an enriched auditory environment during the maternal separation, their circulating CORT levels were partly normalized along with nearly complete rescue of spine density, cortical selectivity, and behavioral accuracy. The rescue was associated with increases in BDNF and reduced expression of a histone that represses BDNF transcription, suggesting that the rescue was driven by epigenetic effects. Because CORT levels were not fully normalized, enrichment may have normalized development rather than reducing stress. This is consistent with studies that have revealed epigenetic involvement with plasticity in sensory systems, and illustrates the interplay of ELS with developmental plasticity [263, 264].

Animal studies indicate that ELS may also induce effects at the auditory periphery. The cochlea expresses a signaling system that is molecularly equivalent to the HPA axis, including receptors to corticosteroids (reviewed in ref. [265]). This suggests that the cochlea may be affected by ELS, particularly early in life during heightened plasticity that occurs prior to maturation. Based on wave I of auditory brainstem responses (ABR), ELS caused reduced responses to short gaps in sound at the level of the auditory nerve [254]. Interestingly, in unmanipulated rats, circulating levels of the stress hormone corticosterone were negatively correlated with both wave I ABR amplitude and synaptic ribbons in inner hair cells [266]. Thus, as a result of ELS, it is possible that altered levels of corticosterone arising from dysregulation of the HPA axis may affect synaptic transmission between hair cells and auditory nerve fibers.

In summary, animal studies of ELS on sensory systems are revealing that ELS induction involving threat, deprivation and/or unpredictability impairs sensory function in visual, somatosensory, and auditory modalities, and alters neural elements in primary sensory brain regions. ELS may alter neural plasticity in sensory regions via shifting critical period elements and excitatory-inhibitory balance, epigenetic regulation of a variety of molecular elements, and downstream effects resulting from HPA dysregulation. These effects vary depending on induction parameters including stress type, duration, intensity, and age of induction relative to developmental windows of susceptibility for specific neural regions. While this calls for common methodologies to allow comparison across studies, different brain regions have different susceptibility across development based on their maturational trajectories. Even studies of maltreatment in children found differential effects on specific brain regions depending on the ages during which the maltreatment occurred [234]. As time windows of stress during development will inevitably vary in children, studying a range of time windows for stress induction in animal models will help elucidate when various brain regions and behavioral consequences are most at risk.

## INTERACTIONS OF EARLY-LIFE HEARING LOSS AND STRESS

Hearing loss maximally impairs ACx encoding and auditory perception early in life during critical periods. Studies in rodents demonstrate that the greatest susceptibility occurs during the critical period for ACx maturation of intrinsic and synaptic response properites [5, 18, 267]. As detailed above for critical periods in general, the opening, maintenance, and closure of the ACx critical period are characterized by shifts in multiple molecular elements including excitatory-inhibitory balance, PV neurons, PNNs, and BDNF. Sensory deprivation from early hearing loss alters each of these elements in ACx, delaying this maturational window for ACx [223, 224, 268–271].

**Fig. 2:**
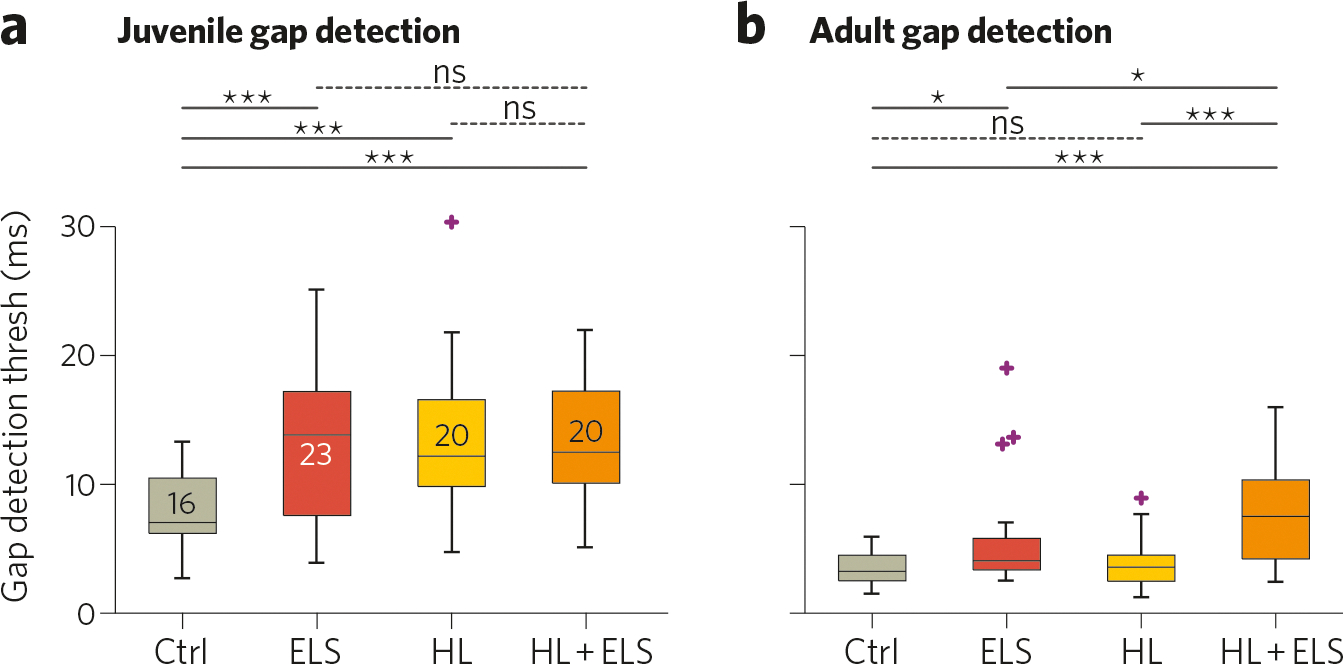
ELS degrades behavioral gap detection, and in adulthood worsens hearing loss-induced deficits. **a** Juvenile Mongolian gerbils were tested between P33–39. **b** Animals were retested as adults (P83–89). Mann–Whitney U test with Benjamini–Hochberg corrections: *p < 0.05; ***p < 0.0002. *Gray numbers* are group n’s. In boxplots, *box edges* are 25^th^ and 75^th^ percentiles; *whiskers* extend to the most extreme data points excluding outliers; *gray crosses* are outliers; *error bars* show SEM.

Early-life stress also affects many of these elements in auditory cortex, as described above. This raises the possibility that a double-hit of early stress and hearing loss may more strongly impact critical period mechanisms to induce particularly detrimental effects on auditory cortical processing and perceptual abilities (Fig. 1, *Elements 1 and 3, magenta and black lines*). To determine whether this combination impacted auditory processing, we evaluated the interaction of ELS and early hearing loss in Mongolian gerbils. Gerbil pups were treated from P11–23 with either maternal separation, earplugs to introduce transient conductive hearing loss, or both (as in refs. [4, 254]). Animals were tested for their behavioral sensitivity to detect short gaps in background noise using gap-inhibition of the acoustic startle (GPIAS; as in ref. [254]). When tested as juveniles, all treatment groups had impaired gap detection compared with controls, but the combination of ELS and hearing loss was no more detrimental than either insult alone (Fig. 2a). Animals were then retested in adulthood. Gap detection is known to mature gradually [255, 272–275]; thus when tested as adults, all groups had better sensitivity to gaps than as juveniles (Fig. 2b). At this age ELS animals were still worse than controls. However an interaction emerged: by adulthood, ELS and early hearing loss impaired gap detection more than either treatment alone.

We are currently following up on these behavioral results to examine potential underlying mechanisms driving this supra-summative interaction of stress and hearing loss that emerges in adulthood. These may include shifts in critical period regulatory elements including epigenetic factors, and/or altered CORT receptor distribution affecting neural excitability. Indeed, adult stress increases GR expression in the auditory midbrain and changes response properties in ACx [276, 277]. The double-hit of ELS and hearing loss may also be detrimental in humans, as described in sections above. While most effects have been evaluated in children, human studies should be extended into adulthood to determine whether these insults interact across the lifespan.

Some relationships between hearing loss and stress in the auditory system can be tested in animal models. For example, evidence outlined above indicates that auditory processing issues caused by early hearing loss could exacerbate cognitive, emotional, and academic challenges induced by early adversity (Fig. 1, *Elements 2 and 3, black lines*). Animal models can be used to evaluate whether cognitive processing in animals exposed to ELS is worsened by early HL, compared with non-stressed animals with HL. Another possible interaction is an increase in stress induced by hearing loss (Fig. 1, *Element 2, blue lines*). While it is reasonable to expect that HL may induce stress in children, to our knowledge it has not been tested explicitly in animal models. If animals are less able to interact vocally with conspecifics, this may induce stress, as inappropriate responses to vocal cues could result in attacks or social rejection. These types of experiments can also elucidate the mechanistic interactions of hearing loss and stress.

## PREVENTION, INTERVENTION, AND FUTURE RESEARCH DIRECTIONS

While future studies are needed to elucidate the impact of auditory processing on ELS-induced cognitive and emotional effects, indirect evidence from children in SES environments, and direct evidence from animal studies of ELS point to likely effects of stress on sensory processing, potentially interacting with hearing loss. Thus it is worth considering how to best identify and amelioriate these issues in children.

To minimize potential interactions of early life adversity or stress with hearing loss, a multifaceted approach is necessary. The most effective way to minimize the negative consequences of hearing loss is to provide early detection and appropriate treatment [145]. In recent years, the adoption of universal newborn hearing screenings has improved early identification [70, 111, 144] regardless of SES. Regions that actively implement universal guidelines for follow-up testing from newborn hearing screenings report a reduction in the number of children lost to follow-up [111]. One major remaining issue is that hearing health care is still poorly covered in the United States, both for patients with private and public insurance [278, 279], and reimbursement and coverage for hearing aids and rehabilitation is still poorer for patients with public insurance than patients with private insurance [129, 132, 280]. Other actions that would reduce the loss to follow-up include offering support for young mothers or families with multiple children, increasing facilities in rural communities, providing transportation, and training a sufficient number of pediatric audiologists [128, 129, 132, 280]. In addition, auditory training programs designed to improve perceptual skills in individuals with hearing loss and auditory processing disorders [281, 282] may be helpful in addressing sensory issues in low SES individuals. These programs often focus on improving perception of speech sounds containing rapid transitions, which animal studies have shown to be affected by ELS [254].

Caregiver communication training is another component of a comprehensive plan to prevent and mitigate the combined effects of early life adversity and hearing loss. As previously mentioned, the linguistic environment in low-SES households is often of a lower quality than in higher-SES homes [11, 63–70], perhaps because low-SES families often have lower parental education levels and less knowledge of children’s developmental and linguistic needs [153]. In addition, many caregivers provide inadequate interactive communication to their DHH children [86, 283]. Several intervention programs have been developed to teach communication strategies to caregivers to improve language outcomes in various clinical populations [142, 284–289]. These training programs typically emphasize caregiver use of developmentally appropriate conversations and diverse, complex speech and have been shown to be effective in improving language acquisition for low-SES children [290], DHH children [289], and low-SES, DHH children [142].

Because time windows of stress during development will inevitably vary in children, studying a range of time windows for stress induction in animal models will help elucidate when various brain regions and behavioral consequences are most at risk. Until we understand these interactions, the third major focus of prevention and intervention regarding early life adversity and hearing loss should be mental health care. Preventative mental health interventions should be considered for all DHH children, especially those who also are at higher risk due to lower-SES, caregiver stress, or family history [291, 292]. One approach to reducing long-term effects of early life adversity is to provide early childhood mental health consultation through collaboration between a mental health professional, early childhood provider, and caregiver [293, 294]. Family-based executive function training may also be helpful for DHH children and their families because family-level organization and self-regulation can affect language skills and executive functioning for these children [295–298]. In addition, screening questionnaires for mental health issues and family stress should be regularly given at medical appointments so that any emerging mental health issues are detected and treated as soon as possible [73]. Finally, provision of resources to families of DHH children, especially those with lower-SES, may alleviate caregiver stress and improve child outcomes [63, 83].

Biological manipulations that are effective in rescuing sensory deficits in animal models may eventually become valid techniques for intervention in children. Auditory perceptual deficits induced by early hearing loss in rodents can be fully rescued by preserving inhibition in auditory cortex [299, 300]. Defects in tactile perception in rodents induced by maternal separation were rescued by blocking glucocorticoid receptors during stress induction [241]. Other approaches that have been successful in ameliorating stress-induced behavioral dysfunction in animals include compensating for blunted or enhanced CORT responses by corticosterone administration or antagonism, modifying neural activity via pharmacology, or upregulating BDNF activity [165, 301–303]. While such approaches are currently aspirational, animal studies that clarify neural mechanisms may facilitate the creation of biological interventions, leading to translational approaches to help individuals with hearing loss who are especially vulnerable due to additional stressors.
